# The Field of Science

**Published:** 1893-10-07

**Authors:** 


### THE FIELD OF SCIENCE.

The *Boston Medical and Surgical Journal* reports a new order regarding the conveyance for scientific purposes of disease germs through the medium of the American post. The Postmaster-General has made a ruling that all disease germs, howsoever carefully sealed, are not in the future to be received for transit, but will be classed with unmailable articles.

Much has been said lately concerning the infection conveyed through flies. Cats now stand arraigned on a similar charge, as offenders equally unwitting and equally dangerous. Quite recently a household was laid low with scarlet fever, due to the contagion brought by a domestic pet which had been in the room of its owner who was recovering, but whose skin was peeling after an attack of fever. Diphtheria and small-pox have also been spread through cats in this manner, and it would be well for the community at large if persons affected with contagious diseases would keep these particular disseminators of infection clear of their presence.

The existence of the disease beri-beri, peculiar to Japan and Java, was formerly ascribed as being due to the anæmic tendencies of the people, as also to excessive supplies of rice as food. Dr. Ashmead who formerly practised many years amongst the natives in Yokohama and Tokio, and made this complaint a special study, is now rather of opinion that it is due to the effect of the carbonic acid which is constantly exhumed from the volcanic soil of certain parts of the two countries. As an antidote for this mountain and sea air are recommended, and Fuji is fast becoming a resort of a curative character during two or three months in the year for patients suffering from this particular disease.

An examination was recently made on the eyesight of native children studying in European schools at Calcutta, with the result that at one large girls' school over 40 per cent. of the children were found to be suffering from defective vision. Our faith in Indian eye-doctors has been somewhat roughly shaken within the last few weeks, but this particular case is not a sequence of recent disclosures concerning native specialist's work. In the case of the Calcutta school children, the defections of their sight were of a sufficiently pronounced type to warrant an examination, the result being as stated above. The only reason which could in any manner account for this state of things seems to be the excessive amount of book-work required by the code, especially in the higher standards. If this be so, the advantages of an European education are paid for at a high price by the children, and it would be well if the school authorities could alter a course of study, which requires long hours of stooping over desks, reading close print in the heat of an Indian summer, when more lectures and practical object lessons would be of even greater educational value.

A weekly contemporary points out the excellent use to which alcohol can be put when applied as a dressing for excoriated surfaces, ulcers, sores, &c. It also has equally beneficial results as an application on bruises, and in cases of violent bruising round or near the eye, if employed as a medication, alcohol effectually prevents the subsequent discoloration of the affected part.

In spite of much evidence brought to bear on the fact that pneumonia, now rife amongst us, owes its existence to hygienic conditions, it is strange that we are content to dwell on the theory and not act upon the knowledge. For the spread of consumption, the scourge of the working classes, is due to a great extent to insanitary surroundings and the ill ventilation of dwelling places. Civilisation to savage nations means a speedy death; the change from a free outdoor life to existence in a confined atmosphere does a sure and speedy work. Very much the same thing is happening daily around us, though in a less marked but equally insidious manner. The ventilation of the cottage home is the last consideration of its designer or its owner, and windows are so constructed that light and air contend alike in vain for entrance. It needs, indeed, insistent repetition before it will be believed that the cases of phthisis in the main are due to breathing air in a confined space which has been breathed again and again, and which contains, therefore, in place of natural life-giving qualities, products alone of combustion and emanations from the body itself.

In the country philanthropists turn their attention nowadays as a matter of course to providing physical recreation for the male community, being by experience convinced that healthy minds and bodies are thereby engendered and maintained. If in the country the due exercise of muscle is recognised as essential, how far more is it necessary for dwellers in the metropolis and other crowded towns. It is unnecessary here to point out why that is so to our readers, who will one and all admit the fact as true. Being so, here lies a wide field for philanthropists. At first sight the difficulty of providing suitable exercise of a recreative nature for the older men in our cities seems almost insurmountable. But it is not really so. A little energy, and bowls, skittles, and like games are all feasible. For fine weather a good yard should be procured, at the end of which or on one side a cheaply covered shed could be erected to provide shelter and sport in bad weather, and this could be lighted for evening use. Some of the money now spent in the cure of illness might thus usefully be spent in its prevention and a sounder population in mind and body might safely be predicted.

